# Letter to the editor regarding “Evaluation of a telemedicine-based training for final-year medical students including simulated patient consultations, documentation, and case presentation”

**DOI:** 10.3205/zma001510

**Published:** 2021-11-15

**Authors:** Malik Majeed

**Affiliations:** 1 University of Cambridge, Addenbrooke's Hospital, School of Clinical Medicine, Cambridge, United Kingdom

**Keywords:** medical education, COVID-19, coronavirus, pandemic, online teaching

## Letter to the editor

Dear Editor,

I read with interest the article by Harendza et al., which reviews the use of telemedicine-based training as a learning tool for medical students whose education has been negatively impacted by the COVID pandemic [1]. As a current medical student in the United Kingdom, our lectures are now exclusively delivered online, and clinical opportunities within both primary and secondary care have been reduced and adapted. This experience, and the current academic literature, are drawn upon when writing this letter. 

The swift introduction of telemedicine-based training and the positive feedback from the students in this study has been impressive. However, it is worth commenting on the potential ambiguity of the Likert scale used in the study. Respectively, scores 2 through 4 on the scale corresponded to “somewhat applies”, “partly applies”, and “rather applies”. Instead, using a scale ranging from “strongly disagree” to “strongly agree” may help reduce ambiguity and therefore more accurately reflect student responses and results interpretation in future studies. 

Secondly, it would be interesting to read the results from the patient questionnaire mentioned in this study. Darnton (2020) found that telemedicine-based consultations negatively impacted medical students’ rapport with patients, citing reduced non-verbal communication and awkwardness arising when asking patients to repeat sensitive information following technical glitches [2]. The patient questionnaire results would be highly useful in assessing any impact – which is especially important when evaluating the viability of a more permanent shift to remote learning post-pandemic.

Finally, I invite the authors’ opinions on recording Zoom sessions. One points to research by Dow et al. in which students benefitted from reviewing pre-recorded “real” consultations in general practice [3]. Perhaps after attempting the telemedicine-based simulation training, students could review a high-level example of a pre-recorded patient-doctor consultation discussing the same patient, which would serve as a good reference for students. In the ‘Clinical Communications Skills’ course at Cambridge, video capture and post-session playback have proven to be a highly useful adjunct to experience within the clinical setting.

The COVID-19 pandemic has increased the need for, and accelerated the transition toward, innovative, remote learning strategies in medical education. The research by Harendza et al. is both important and necessary in evaluating these novel education methods. 

## Competing interests

The author declares that he has no competing interests.
